# Eco-evolutionary dynamics, coding structure and the information threshold

**DOI:** 10.1186/1471-2148-10-361

**Published:** 2010-11-24

**Authors:** Folkert K de Boer, Paulien Hogeweg

**Affiliations:** 1 Theoretical Biology and Bioinformatics, Utrecht University, Padualaan 8, 3584 CH, Utrecht, Netherlands

## Abstract

**Background:**

The amount of information that can be maintained in an evolutionary system of replicators is limited by genome length, the number of errors during replication (mutation rate) and various external factors that influence the selection pressure. To date, this phenomenon, known as the information threshold, has been studied (both genotypically and phenotypically) in a constant environment and with respect to maintenance (as opposed to accumulation) of information. Here we take a broader perspective on this problem by studying the accumulation of information in an ecosystem, given an evolvable coding structure. Moreover, our setup allows for individual based as well as ecosystem based solutions. That is, all functions can be performed by individual replicators, or complementing functions can be performed by different replicators. In this setup, where both the ecosystem and the individual genomes can evolve their structure, we study how populations cope with high mutation rates and accordingly how the information threshold might be alleviated.

**Results:**

We observe that the first response to increased mutation rates is a change in coding structure. At moderate mutation rates evolution leads to longer genomes with a higher diversity than at high mutation rates. Thus, counter-intuitively, at higher mutation rates diversity is reduced and the efficacy of the evolutionary process is decreased. Therefore, moderate mutation rates allow for more degrees of freedom in exploring genotype space during the evolutionary trajectory, facilitating the emergence of solutions. When an individual based solution cannot be attained due to high mutation rates, spatial structuring of the ecosystem can accommodate the evolution of ecosystem based solutions.

**Conclusions:**

We conclude that the evolutionary freedom (eg. the number of genotypes that can be reached by evolution) is increasingly restricted by higher mutation rates. In the case of such severe mutation rates that an individual based solution cannot be evolved, the ecosystem can take over and still process the required information forming ecosystem based solutions. We provide a proof of principle for species fulfilling the different roles in an ecosystem when single replicators can no longer cope with all functions simultaneously. This could be a first step in crossing the information threshold.

## Background

The information threshold [1] puts a limit on the maximum amount of information that can be evolutionarily maintained by a single population of replicators. For an evolutionary process this implies that the length of genomes of replicators and the number of errors during replication (mutation rate) is limited. It raises the question of how a 'simple' prebiotic system can evolve towards a more complex living system. To increase the complexity of a prebiotic system, replicators which should both store information and act as an enzyme, must have been able to accumulate and pass on information correctly. To correctly transfer more and more genetic information between generations, the fidelity of replication has to improve as well. This could be done for example with specific replicase or proofreading enzymes. However, this requires an increased coding length, which cannot be maintained without these same enzymes. Thus it is not possible to have a (large) genome without enzymes, but the evolution of enzymes would not be possible without large genomes. This is referred to as Eigen's paradox [2,3].

This paper aims to extend the context in which the information threshold is studied and its possible role in the early evolution of life. Traditionally the information threshold is formulated in terms of a master and quasispecies of genotypes with a static, single-peaked fitness landscape [1]. These studies have been extended by taking a nonlinear genotype-phenotype mapping into account, using RNA folding as a prototype example. In such systems neutral mutations play an important role, and due to this neutrality larger sequences can be maintained, but this increase is limited [4,5]. We extend these previous studies in three different directions: 1) Replicators have a flexible coding structure, leading to a variable genome length and variable genotype-phenotype mapping, (2) the acquisition, rather than the maintenance of information is studied, and (3) replicators evolve within a non static environment.

The first attempt that has been proposed to cross the information threshold involved the introduction of multiple replicators [6]. Such hypothetical replicators, later on mostly considered to be RNA (or another catalyst), could form the basis of a prebiotic ecosystem, sometimes referred to as 'the RNA-world' [7]. Most likely, the only viable solution to Eigen's paradox lies in the co-existence of several different replicators, such that the information necessary for coding enzymes can be stored and transmitted by a population of co-existing smaller replicators. Due to the fact that the co-existence of different species is typically considered to be an ecological problem, these approaches have been called 'the ecological solution' [8]. The two main models that attempt to formulate such ecological solutions to Eigen's paradox are the hypercycle model [6,9,10] and the metabolic system model [8,11,12]. However, these studies address the maintenance of a replicator-ecosystem despite mutations (or stability against invasion of parasite mutants), rather than the generation of an ecosystem *as a consequence *of high mutation rates. Moreover, although the original question involved the mechanism of obtaining functionality despite high mutation rates, no function beyond reproduction was incorporated in these models. Here we study how a system can cope with externally defined requirements under various mutation rates (per base). We consider the case that viable replicators can evolve functionality through either individual or population based diversity [13-15]- eg. all replicators perform all functions by themselves (like a Swiss army pocket knife) or different functions are divided over different replicators, the latter being an ecosystem based solution. In other words, the ecosystem as a whole can provide a solution for the posed 'problems' in the environment. In our system these problems in the environment change over time, co-evolving with the replicators, resulting in a dynamic fitness landscape.

Regarding this environment, almost all theoretical studies published so far have demonstrated that some kind of spatial structure is indispensable for the persistence and/or the parasite resistance of any feasible replicator system [8,9,11]. Through spatial pattern formation, selection is extended from purely individual-level selection to multi-level selection. Multi-level selection is considered to be a defining property of ecosystems [9,10] and the success of evolution strongly depends on how the ecosystem is able to structure itself [16,17]. As such, our model system allows for an emergent structure on two levels: both the coding of replicators and the spatial distribution within the ecosystem.

To summarize, we study the problem of the information threshold, bearing in mind that information has to be *obtained*, rather than merely *maintained *under high mutation rates. We use a flexible coding structure at the level of individuals and in addition we allow for the evolution of ecosystem based solutions using a spatial co-evolutionary setup. We study how such a system copes with high mutation rates, ie. whether an ecosystem based solution can replace an individual based solution when the latter is not attained.

## Results

We study the information threshold assuming that all replicators are under physiological constraints that require the acquisition of mechanisms (or path-ways) to process food for survival and reproduction. Replicators have to cope with their varying (co-evolving) environment, having different solutions for different situations (prey). To this end we use co-evolutionary function approximation as a tool for modeling eco-evolutionary dynamics [15,17,18]. We develop a model-ecosystem of predators, prey and scavengers. Predators and scavengers are the studied replicators and the consumption of prey acts as an analogy for coping with the environment. The environment takes the form of co-evolving numerical examples or problems (prey). Prey consists of numerical values, here (x, y). During prey replication, these values can change by mutation. Coping with the environment (eating prey) is defined as producing a numerical value defined by a global external function on the values of a prey (for example if the global function would be simply addition, then a predator producing '5' for prey (x = 2, y = 3) would get maximum fitness).

**Figure 1:**
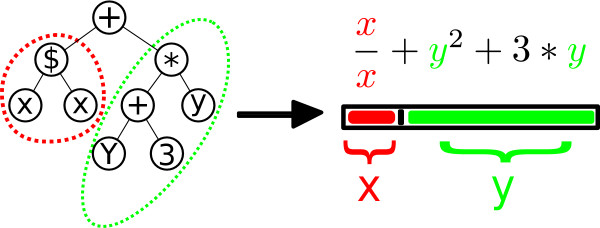
**Coding Structure**. The genetic representation of predators and scavengers is a tree-like coding structure. This genotype defines the phenotypic reaction to a prey, based on the (x, y)-value of this prey. That is, how does one process prey consisting of a certain amount of nutrients, x and y. The functional representation of this replicator would be (+ ($ x x) (* (+ y 3) y)).

The genotypes of predators and scavengers are based on LISP-constructs, which allow for a flexible integration of numerical functions in a genome (see Figure 1 and methods section).

**Figure 2:**
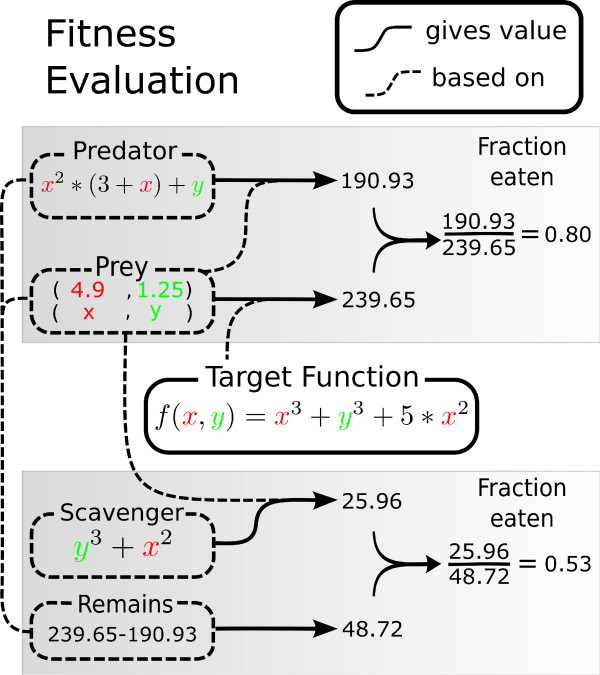
**Fitness Evaluation**. Schematic representation of fitness evaluation of predators, scavengers and prey. Dashed lines denote on basis of which value a response is. That is, predators produce a value based on the (x, y)-values of a prey (colored red and green respectively). This value, relative to the value which the prey produces (based on the evolutionary target), defines the fraction of prey which is eaten by the predator. A scavenger feeds on the remains of prey, based on the same (x, y)-values of prey. Fitness is based on the fraction of prey which is eaten. In this particular example, the fitness of this prey would then be 0.2 (1. - 0.8) and the predator and scavenger would get respectively 0.82 (*e*^-0.2^) and 0.63 (*e*^-0.47^) added to their fitness.

Predators and scavengers observe the state variables (x, y) of prey and should respond by producing the numeric value in accordance with an externally defined function (see Figure 2). For predators this is the exact value of the target function; for scavengers this is the value of what is left over by the predator. Note that scavengers do not observe what has been eaten by predators. Each generation a replicator is confronted with several prey competing with surrounding replicators (only of the same kind) to eat it and fitness is defined proportional to the fraction of prey consumed.

The evolutionary targets used are chosen such that a solution is evolved easily by predators alone under a wide range of parameters. They differ in the absolute minimum amount of coding needed and in the composition of x, y and mixed (x, y)-terms. The amount of coding is expressed in the number of elements (i.e. operator, variable or constant) on a genome, referred to as *length*. Table 1 lists the evolutionary targets used, the corresponding minimal coding length *m*, and some examples of genomes with minimal coding length for a solution. Note that this minimum can only be reached by 'smart' coding. That is, coding used for different terms has to overlap. An example of overlapping terms is (* 2 (+ x y)) (coding length 5), which is shorter than (+ (* 2 x) (* 2 y)), which uses 7 elements to code for the same function.

**Table 1:** Evolutionary Targets

	*m*	Evolutionary Target	Minimal Coding Example
(a)	13	*f*(*x*, *y*) = *x*^3 ^+ *y*^3 ^+ 5*x*^2^	(+ (* (* (+ x 5) x) x) (* (* y y) y))
(b)	15	*f*(*x*, *y*) = *x*^3 ^+ *y*^3 ^+ 5*x*^2 ^+ *xy*	(+ (* (+ (* y y) x) y) (* (* (+ 5 x) x) x))
(c)	15	*f*(*x*, *y*) = *x*^3 ^+ *y*^3 ^+ 5*x*^2 ^+ 2*y*^2^	(+ (* x (* (+ 5 x) x)) (* (* y (+ 2 y)) y))
(d)	19	*f*(*x*, *y*) = *y*^4 ^+ *x*^3 ^+ *y*^3 ^+ *yx*^2 ^+ *y*^2^	(+ (* (* x x) (+ x y)) (* (* (+ (+ y 1) (* y y)) y) y))

Evolutionary targets with corresponding minimal coding length *m *needed to code for them. In the last column an example of a genome with the minimal length coding for such a full solution.

The coding and the setup of our model-ecosystem enables the possibility to find two types of solutions: individual based solutions where all possible prey can be fully consumed by a single predator and an ecosystem based solution where a solution is formed by an ecosystem of multiple replicators, namely a predator and a scavenger. Simulations can be classified into three main classes: an individual based solution whereby the majority of prey are fully consumed by single predators coding for the full target function, an ecosystem based solution whereby the majority of prey are fully consumed by complementary predator-scavenger pairs which together code for exactly the target function, or no solution at all when none or only a small minority of prey is fully consumed (by predators or predator-scavenger pairs which do not code for the whole target function). Note that an individual based solution excludes an ecosystem based solution. However, it is possible that an individual based solution replaces an ecosystem based solution over evolutionary time.

**Figure 3:**
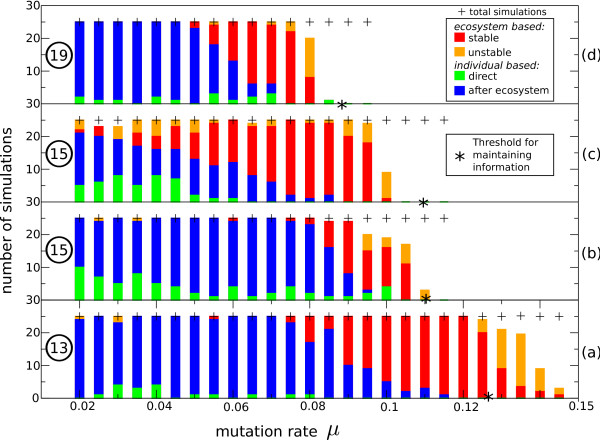
**Evolved Solutions under Different Mutation Rates**. Type of solutions (classified as described in the methods section) under different mutation rates (per base) for the evolutionary targets of table 1 with minimal coding length of (a) 13, (b) 15, (c) 15, (d) 19. Note that the exact nature of the target does also make a difference as shown by the difference in shift for both targets with a minimal coding length of 15. Blue and green represent simulations which evolve an individual solution, where blue has a transient state of ecosystem based solutions. Red and orange represent ecosystem based solutions, where in the orange cases this solution is lost again later in evolution. For each target the information threshold for maintaining the target is indicated with a star. Above this mutation rate the shortest solution for this target cannot be maintained as described in the last paragraph of the results section.

In Figure 3 we see a clear transition between the type of solutions found under various mutation rates. A similar pattern is seen for all functions in table 1. The shift to the right occurs because of the different length of coding needed. That is, under lower mutation rates an individual based solution is evolved in almost all cases (blue and green) and under increased mutation rates it becomes increasingly difficult to reach an individual based solution. At the solutions increase (orange and red). These ecosystem based solutions can be found up until quite severe mutation rates, clearly beyond the range of individual based solutions. The exact transition is also influenced by the nature of coding needed for a target, as exemplified by the difference in transition for targets with minimal coding length 15.

The remainder of this section is divided in two parts. First we will discuss the role of mutation rates on the information accumulation of individuals by focusing on basic genomic characteristics such as length and structure. Secondly, we extend our scope by looking at the role of co-evolutionary and ecosystem dynamics and how an ecosystem based solution can arise under circumstances where individual based solutions cannot. With these results we show how flexibility in a (co-)evolutionary system can help in overcoming the information threshold.

### Information Accumulation and Individual Based Solutions

**Figure 4:**
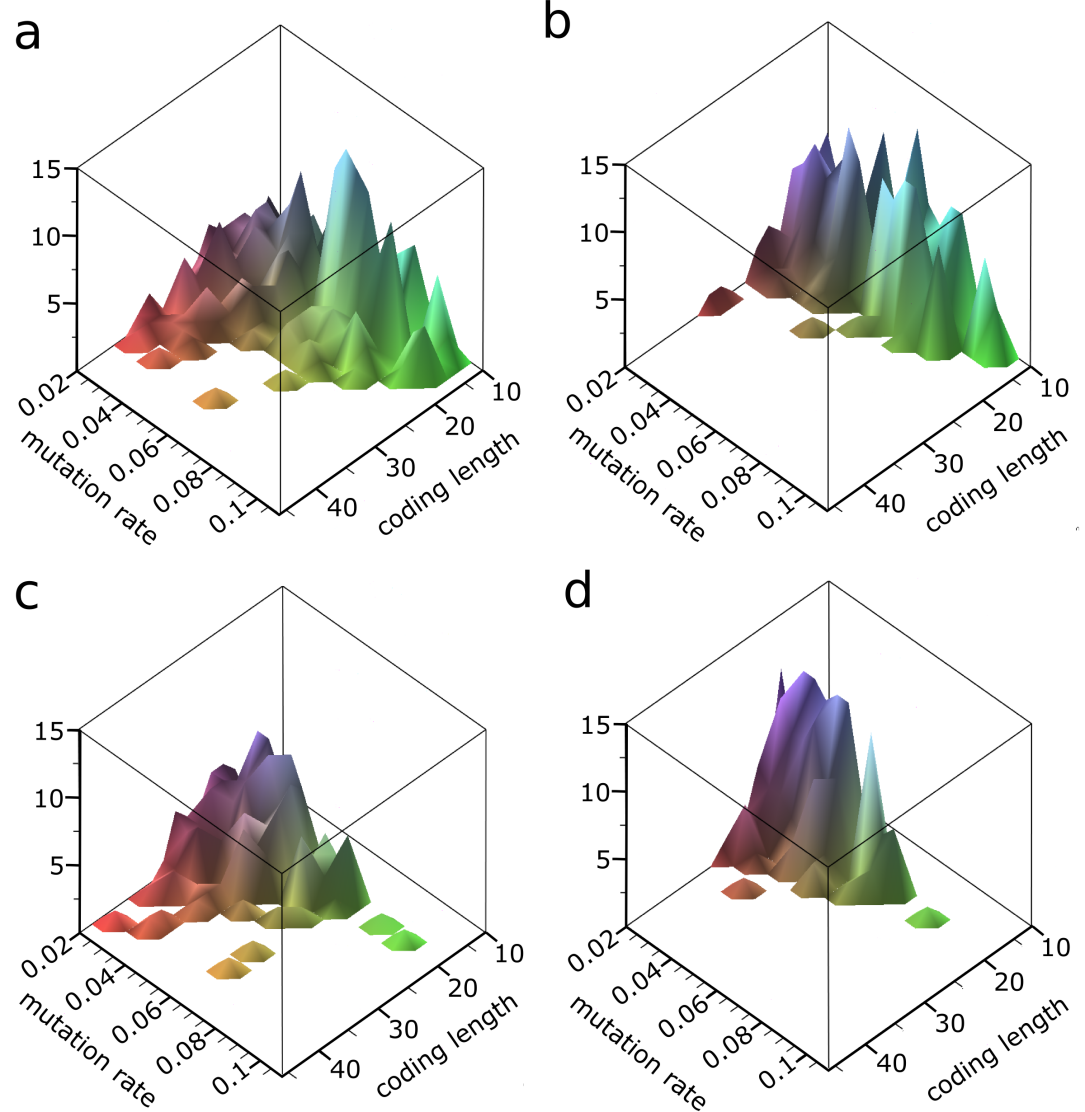
**Initial and Final Coding Length for Different Mutation Rates**. A decrease in initial coding length under higher mutation rates is observed. Restructuring of initial solutions after prolonged evolution also decreases length. For each mutation rate 25 simulations are run. The length distributions under different mutation rates are shown for (a) first evolved individual base solutions and (b) most compact individual based solution 250 generations later, for the target with minimal coding length 13; (c) first evolved individual based solutions and (d) most compact individual based solution 250 generations later, for the target with minimal coding length 19. Note that some first evolved solutions are lost from the population after prolonged evolution due to the information threshold (for example the solution found for the longest target with *μ *= 0.095 in (c) is lost in (d)).

First we focus on the full solution of individual replicators, and therefore we study the evolutionary trajectory from the point in time where such a solution arises in the predator population. Figure 4 shows the influence of mutation rates on the length of evolved solutions for the shortest and longest evolutionary targets, respectively. For each mutation rate 25 different simulations are run. The distribution of the length for the full solutions, evolved under different mutation rates is shown. Note that in some cases the correct individual based solution first reached cannot be maintained due to the information threshold and is lost again. The initial genome length of the predators first to reach the evolutionary target decreases under increasing mutation rates. Secondly, it is evident that 250 generations after the arrival of this first solution, the length of coding used by the solution becoming dominant in the population has decreased. The length of coding approaches the absolute minimum for the corresponding targets. Thus the predators in the ecosystem have restructured their coding such that the same phenotype is coded for by a shorter genome. Such shorter genomes tend to be more robust because of less mutations per generation. In table 2 an example of this streamlining of genotypes and altered genotype-phenotype mapping is shown for *μ *= 0.03. After arrival of the first solution, multiple 'mutant' strains (not necessarily ancestors) with the correct individual solution arise in the ecosystem, all with a shorter coding for the same phenotype. These different genotypes co-exist in the population, however in the long term, the most compact coded solutions will out-compete those with a longer genome.

**Table 2:** Streamlining of Individual Based Solution

25: (- (* (* y y) y) (* (- (- x x) x) (+ x (* (+ 3 (- 1 (- (- x x) x))) x))))	
21: (- (* (* y y) y) (* (- (- x x) x) (+ x (* (+ 3 (+ 1 x)) x))))	19: (- (* (* y y) y) (* (- (- x x) x) (+ x (* x (+ 4 x)))))
17: (+ (* (* y y) y) (* x (+ x (* x (+ 3 (+ x 1))))))	15: (+ (* (* y y) y) (* x (+ x (* x (+ 4 x)))))

Observed streamlining of genotypes for the phenotype of an individual based solution found in a simulation with *μ *= 0.03. After the arrival of a first individual based solution of length 25, the length of consecutive mutants is decreased after prolonged evolution. Examples are shown of two strains leading to a solution with length 15 and 17 respectively. Note that intermediate mutants are not necessarily shown.

These observations strongly suggest that, despite a strong preference for having a coding structure as short as possible, predators initially exploit more information than strictly necessary to evolve a solution. Under increased mutation rates these longer solutions cannot arise or maintained anymore. Thus we observe that high mutation rates decrease the degrees of freedom and thereby restrict the chance of finding an individual based solution. Under severe mutation rates an individual based solution cannot be found anymore at all.

**Figure 5:**
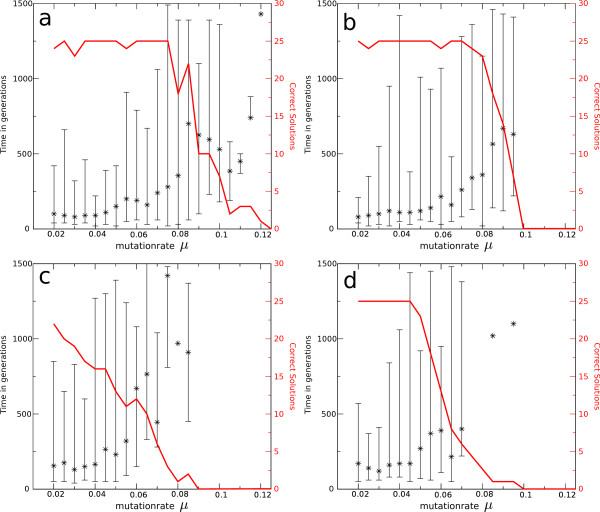
**Generations Needed to Evolve a Full Solution**. For evolutionary targets with minimal coding length (a) 13, (b) 15, (c) 15 and (d) 19, the median number of generations needed for the evolution of a full solution is shown. Error bars depict the minimum and maximum number of generations. On the right (in red) the actual number of solutions out of 25 simulations per mutation rate is plotted. Among the solutions shown are some which cannot be maintained and are lost from the population. Leaving out these solutions even strengthens our conclusions. Prolonged experiments (maximum generations = 20000) with high mutation rates give comparable results. That is, results do not qualitatively depend on the amount of time provided for information accumulation.

These conclusions are corroborated by results on the average time to reach the evolutionary target. Figure 5 illustrates that under increased mutation rates it takes longer to evolve a full solution. This contradicts the expectation that a higher rate of change and a smaller search space because of smaller genomes would lead to a faster coverage of genotype space. As shown in Figure 4 only a subset of genotypes coding for the full solution is reached (depending on coding length). Moreover, on average it takes predators longer to evolve a solution, again suggesting mutational restrictions in usable coding length.

To disentangle the role of mutation rate and genome length, and establish that it is indeed the genome length rather than mutation rates which determine efficacy, we perform experiments with an external restriction on the available genome length for replicators (simulating a lethal mutation for replicators which exceed a certain genome length). As shown in table 3 both under low and high mutation rates it becomes increasingly difficult to find individual based solutions. The median time needed for evolving individual based solutions increases and success rate drops dramatically. Assuming 'optimal' mutation rates [19,20] would predict that predators with a restriction on length would perform better under higher mutation rates in search for an optimal rate of change. However, although the influence of both length and mutation rate cannot be disentangled completely, this is clearly not the case with present results. Taking into account the decreased multiplicity of reachable genotypes coding for individual solutions as observed in Figure 4, we can only conclude that reachable genotype space (and solutions) are restricted by high mutation rates via the genome length of replicators.

**Table 3:** Length Restriction

	∞	13	15
***μ***	**solutions (median time)**		

0.04	25(90)	5(150)	12(168)
0.08	18(355)	8(806)	5(730)

Evolution of replicators with a restriction on their genome length of 13 and 15 elements respectively, compared with no restriction for *μ *= 0.04 and *μ *= 0.08. With restriction, replicators exceeding the maximum number of elements allowed, are considered as lethal mutants. The number of simulations which established an individual based solution are shown and in parentheses the median time to find these solutions. For each set 25 simulations are run with the shortest evolutionary target (minimal coding length = 13).

### Population Based Diversity and Ecosystem Based Solutions

**Figure 6:**
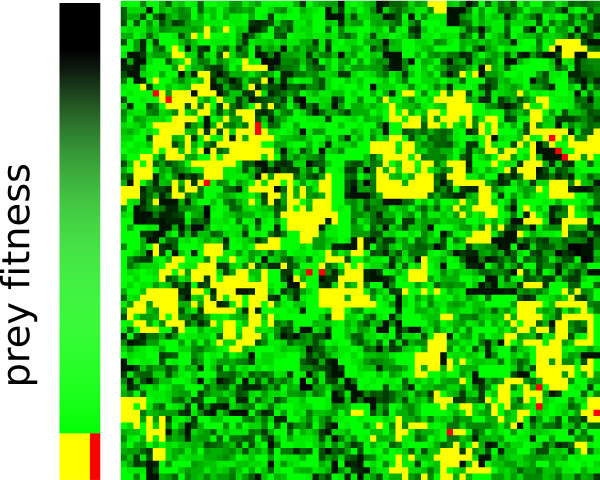
**Spatial Ecosystem Distribution**. This figure shows the spatial structure of an ecosystem based solution under high mutation rates. The shade of green denotes the fitness of prey, or rather: how much of the prey is eaten. Prey depicted as yellow are fully eaten by an ecosystem based solution. Red denotes single prey which are fully eaten by a predator alone (not being an individual based solution). In this case the pattern is governed by the prey which are fully eaten by a predator-scavenger pair. Such a pattern, with comparable numbers of 'yellow' prey, can only be met when a correct ecosystem based solution is present in the population.

Before an individual based solution has been reached, the composition of the predator population is heterogeneous. Due to co-evolutionary dynamics between predator and prey, both populations become speciated. Prey maximize the genotypic distance between the different sub-populations and different sub-populations of predators specialize to-wards each of these (for an extensive analysis of these dynamics, see [15]). Because of the spatial embedding, predators and prey structure them-selves such that wave-like patterns arise as shown in Figure 6. In table 4 an example of co-existing sub-populations is shown, taken from a simulation with the longest evolutionary target at *μ *= 0.075. Within the population of prey, one sub-population evolves a high x-value and a low y-value, while in the other sub-population this is reversed. Predators speciate in 'eating' a different part of prey. One sub-population feeds mostly on y (i.e. contains mostly y-terms), prospering on prey with a high y-value, and a predator feeding best on the x-value of prey has the opposite preference. Note that this does not necessitate strict partitioning in x and y-terms of the evolutionary target.

**Table 4:** Coding Example of Ecosystem Based Solution

predator	scavenger
((* x (* x (+ y x))))	((* y (+ (* y (+ (* y y) y)) y)))
((* (+ (* (+ (* y y) y) y) y) y))	((* (+ y x) (* x x)))

Example of evolved ecosystem based solution for the longest evolutionary target used: *f*(*x*, *y*) = *y*^4 ^+ *x*^3 ^+ *y*^3 ^+ y _* _x^2 ^+ *y*^2^. These predator-scavenger combinations can feed perfectly on all possible prey in the model universe.

Scavengers, feeding on the remains of prey, also speciate during evolution, trying to have a preference opposite to the dominant predator in their neighborhood. Under moderate mutation rates, predators keep evolving towards the full evolutionary target, possibly diminishing the remains of prey more and more. Scavengers can keep up in such a case only by feeding on smaller parts. Note that they can keep fitness, because fitness is assigned as a fraction of the remains. However, when an individual based solution evolves, scavengers loose all their functionality because there is nothing left to feed on.

Only under higher mutation rates, the ecosystem based solutions become a stable evolutionary attractor. Under high mutation rates the system is no longer able to evolve individual based solutions due to mutation rates and the constraints in genome length as shown above. Due to the high mutation rates, predators can obtain only enough information to code just for feeding "sufficiently enough" on local prey. Robustness in solving only a subset of possible prey with high local fitness is 'chosen' above high, but unstable global fitness (meaning the hypothetical fitness they could acquire on all prey). Due to spatial pattern formation several such partial solutions can co-exist in a stable ecosystem. Scavengers are able to feed on the remains of prey, resulting in a structured ecosystem based solution, as shown in Figure 6. Despite the sub-population sizes possibly being small due to a large amount of mutants, predators and scavengers forming the correct complementary partial solutions can stabilize over time, even under high mutation rates, as shown in Figure 3.

Finally we compare our results for acquiring information with the threshold for maintaining information [1,21]. When seeding the population with individual based solutions of the minimal coding length for the evolutionary target, these full solutions can only be sustained for mutation rates up until *μ *= 0.126, *μ *= 0.110 and *μ *= 0.088, respectively for the targets with a length of 13, 15 and 19 elements (shown as stars in Figure 3). Therefore we can conclude that the limits posed by the information threshold are even more severe for obtaining information than they are for maintaining information. Moreover, individual based solutions with a longer genome (coding for the solution) cannot persist under such severe mutation rates and the individual based solutions will be lost completely or recoded to a shorter solution. Thus, by allowing for variable coding, the information threshold for obtaining certain functionality can already be alleviated by using a more compact coding.

**Figure 7:**
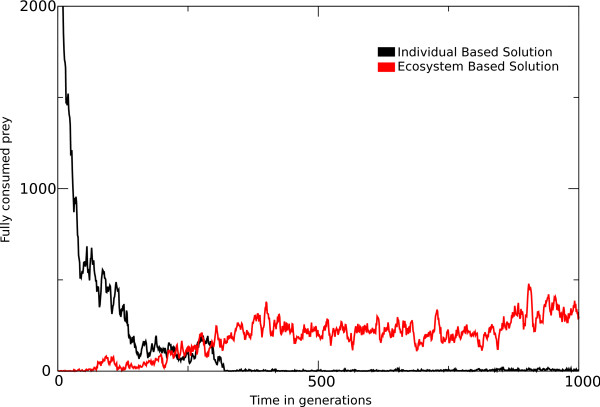
**Passing the Information threshold**. When seeding a population under mutation rates above the information threshold (*μ *= 0.13), with correct individual based solutions, these solutions are quickly lost from the population. This is shown by the declining number of prey which are eaten by correct individual based solutions(black line). The loss of these individual based solutions creates a niche for ecosystem based solutions, which indeed arise as can be observed by the increase of prey consumed by a correct ecosystem based solution (red line).

As for the actual crossing of the information threshold (for maintenance of information), ecosystem based solutions should be able to fully consume prey under mutation rates under which individual based solutions with minimal coding cannot even persist. In Figure 7 we show an example for the shortest target with *μ *= 0.13, i.e. above *μ *= 0.126, identified as the maximum mutation rate under which an individual based solution can be maintained. We observe thus a case where an individual based solution with a most compact coding (13 elements) is indeed out-competed completely, and the ecosystem takes over, processing the required information and still consuming a considerable amount of prey fully each generation.

## Discussion

First we studied the influence of mutation rates on the evolutionary trajectory by observing how evolved individual based solutions are coded for under different mutation rates. We also looked at the structure and length of predators first reaching the evolutionary target. It has previously been observed that the information threshold restricts the amount of information that replicators can maintain under increased mutation rates [1,21]. We showed that under high mutation rates a more severe threshold restricts the required increase of information before replicators are fully functional.

For genomes with a fixed length it has been shown analytically that 'optimal' mutation rates exist [19,20], that mutation rate itself is a selectable trait [22], and that the time to reach a target increases given higher mutation rates [23]. Moreover, for evolving towards a static target with variable genome length it has been shown that high mutation rates lead to compact coding for functionality on a genome [24]. Here we see that in the case of flexible coding, replicators use different lengths under different mutation rates. For a fixed mutation rate per base, more length leads to more mutations per replicator and thus by adjusting the length of coding, the mutation rates per generation are altered. However, a restriction of genome length does not result in a more efficient covering of genotype-space under higher mutation rates nor in an increase in efficacy. In contrast, under lower mutation rates without length restrictions, an increase in the success rate can be related to an higher diversity of reachable genomes coding for individual based solutions. That is, an increased multiplicity of available solutions has a clear positive effect on efficacy. The possibility to generate longer genomes increases efficacy. Similar results have been shown experimentally with the isolation of novel ribozymes from random-sequence RNA pools, where longer random sequences increase the probability of finding complex structures [25]. With variable genome length it is not maximum mutation rates which increase genetic diversity as one would intuitively expect. Instead, moderate mutation rates increase genetic diversity through increasing genome length and therewith enlarges evolutionary search space which in turn maximizes evolutionary efficacy. The diversity of attainable genomes, coding for an individual based solution, decreases under higher mutation rates. However, we have shown that high mutation rates lead to the persistence of more niches for replicators which are only partially functional. In contrast, recent work based on RNA-like replicators has shown that a lowering in mutation rate can lead to an increase of niches [26]. The difference between these systems is that in our case there is a predefined set of "tasks", whereas in a RNA-system the only "task" is (catalytic) reproduction. When selection is solely acting on replication through interaction, high mutation rates disrupt the interaction strength within an ecosystem by dilution of the fittest sequences, preventing the formation of new species. This differs from our current system where high mutation rates maintain more niches by preventing the out-competition of multiple partial solutions (the ecosystem) by the full solution. When not fully functional replicators are viable (i.e. selection is based on functionality instead of replication), replicators can suffice with lower functionality under increased mutation rates. This shows a new side of the information threshold: the impossibility of evolving replicators with full functionality leads to an increase in diversity because of the multiplicity of partly functional replicators in the system. Under moderate mutation rates, spatial structuring of co-evolving populations benefits the information integration over evolutionary time in replicators [16-18]. If, however, the necessary information cannot be integrated in a single replicator, the diversity of partial solutions can be kept in the ecosystem because of this co-evolutionary nature and spatial distribution of the system.

We showed that under high mutation rates our system does switch from individual based solutions to-wards the generation of an ecosystem based solution. Thus we conclude that ecosystem structuring enables the increase of the complexity despite the presence of an information threshold.

## Conclusions

The coding structure of evolved replicators reveals the influence and severity of mutation rates. The information threshold not only influences the maintenance of information in the genome, but it also constrains the degrees of freedom of the evolutionary trajectory by restricting the permissible genome length. In our system, multiple 'solutions' are possible due to a complex genotype-phenotype mapping and freely evolving coding structures. However, the number of genotypes coding for a full solution which can be reached is increasingly restricted by higher mutation rates. If the length of maintainable information is limited by the information threshold, replicators can adapt their coding structure. In this way, for a given functionality the information threshold can partly be alleviated by using a different, more compact, coding. This aspect of the information threshold is of great importance for questions about the evolution of complexity. We show that in a system with such severe mutation rates that an individual based solution cannot evolve, the ecosystem can take over and still process the required information, forming ecosystem based solutions. Therefore, we conclude that, when taking eco-evolutionary dynamics and flexible coding structures into account, the integration of information within the ecosystem under circumstances where individual based solutions cannot evolve, can be a feasible solution to Eigen's paradox and a possible option for crossing the threshold for obtaining information.

## Methods

We use a stochastic Cellular Automata (CA) model, which is a spatially extended, synchronously up-dated individual-based simulation model. It consists of (vertically stacked) two-dimensional square grids on which individuals are located. The grids are made up of 75 × 75 cells, and the boundaries are toroidal. Simulations not displayed here show that the qualitative behavior of the system does not depend on the size of the grid if larger than 50 × 50, which is large enough for spatial patterns to develop. One square in a grid, hereafter called a 'cell', holds at most one individual. The different grids are located exactly on top of each other, as if each location (*i*, *j*) holds three individuals. Interactions between individuals within the same grid as well as interactions between individuals from different grids are all local in a 3 × 3 neighborhood. That is, a neighborhood consists of the eight cells adjacent to a cell and the cell itself (Moore neighbors). Our model distinguishes three types of individuals, with each type located on a separate grid. The state of the model system is fully specified by the type, the state and location of all individuals. The three different types are called prey, predators and scavengers. The state of predators and scavengers is the numerical function they encode and the state of prey is a numerical (x, y)-value. Fitness of predators and prey depend on their co-evolutionary relationship and scavengers feed on the remains of prey, only after the predators have finished. Thus, scavengers have no influence on the evolutionary pressures of predators and prey. Scavengers are only implicitly evaluated on how well they complement predators and only see the original values of prey. Therefore scavengers cannot see how much of a prey has already been consumed by a predator.

We use a system-wide defined evolutionary target, as carried out in function approximation methods [15,17,18]. In our case this is a numerical function considered in a limited domain only, namely *x *= 0.0, 5.0 and *y *= 0.0, 5.0. Table 1 lists the evolutionary targets used, the corresponding minimal coding length *m*, and some examples of a 'genome' coding for a solution.

Prey consist of genotypes which are instances of (x, y)-value pairs within the function domain. That is, each prey represents a certain problem which has to be solved by the predators. This value-pair maps via the predefined target function to an unique value, *f *(*x*, *y*), which can be considered as the solution to the particular problem presented by a prey. This unique value has to be matched by predators, which determines how much of this prey is consumed (see Figure 2). Every time step each of the values of the prey population is subject to a 40% chance on mutation per value. Both the values of (*x*, *y*) of a prey can change randomly between the defined mutational boundaries (that is, current value plus or minus 0.2). The genotype space of prey (domain of function landscape) is not toroidal. If an x or y-value of a prey is on the border of the domain, it can only mutate in one direction (to keep mutation rate constant, mutations over the border will not be neglected, but reflected).

Predators and scavengers have the same genomic architecture. The genetic representation is in the form of a program (i.e., a functional representation, as in genetic programming). Many different programs can code for the same numerical function. The genome consists of a limited set of terminals and operators, based on LISP-programming, coding for a function (for an example see Figure 1). The set of operators is {+,-, x,$}, where $ is a save division operator, often used in genetic programming, which gives 1 when dividing by zero. The set of terminals is {x, y, C}; x and y are the variables and C is a constant defined at declaration either as an integer between 0 and 10, or as a float between 0 and 1.

Both predators and scavengers selected after evaluation are subject to point mutation, using a mutation rate *μ *per element and a gross chromosomal rearrangements(GCR) rate per genome. Due to the treelike representation of genomes, it is important to realize that mutating elements high in the hierarchy can possibly affect underlying elements. When a mutation leads to the change of an operator into a terminal (either a variable or constant), underlying elements are discarded. In the reversed case (terminal mutating into an operator) a random sub-tree of maximal 3 elements is added. In all other cases only the element itself mutates and the under-lying elements remainfluntouched. A gross chromosomal rearrangement-event (chance of *μ_GCR_*) means that a randomly chosen part of a genome is overwritten with another randomly chosen (possibly overlap-ping) part of this genome. Where point mutations can only lead to a gradual increase or decrease in length, GCR can possibly lead to a sudden large increase in length. However, although GCR speeds up the process, even without GCR qualitatively similar results are obtained. Results are only shown for *μ_GCR _*= 0.1 and *μ_prey _*= 0.4, however test simulations have shown that qualitative results do not depend on these parameters. In most simulations we are interested in *μ*, which is varied between 0.02 and 0.15. Note that under neutral expectations there is a small bias for predators and scavengers to become smaller, due to the combination of mutational operators and treelike representation (it is easier to loose a large sub-tree, than to gain it). However, this bias is the same for all different *μ *and of no influence for the results shown.

Simulations start with a population of 5625 (one individual per cell) for each type of individual. Prey start with a random (x, y)-value pair within the domain. Predators and scavengers start with a random generated genome with an average length of 13.8. The consecutive application of a simple algorithm on all positions of the spatial grids (of which each position i, j contains a prey, a predator and a scavenger respectively) defines the temporal dynamics of the model. All individuals are replaced every time step (leaving no empty grid cells), keeping population sizes constant. Asynchronous simulations with overlapping generations for predators and scavengers give qualitatively the same results. The synchronous algorithm used runs as follows:

### • evaluation

1. check for the prey at position (i, j), which local predator approximates f(x, y) best and may feed on this prey.

2. if there is some of the prey left → check which local scavenger is most suited to feed on the remains.

3. define fitness for all types of individuals. In order to keep a clear evolutionary signal, individuals whose approximation are identical within its neighborhood, acquire the same fitness for this prey. (see Figure 2 for an schematic representation of the fitness evaluation).

- Prey fitness is the fraction of it which has not been eaten by the predator.

- Predator fitness consists of the sum of the fraction of prey it eats in a local neighborhood. Each consumed prey adds *e^- ^*^(1-*fractioneaten*) ^to the fitness of the predator. This makes *f *= 9.0 the maximum fitness when all prey in the neighborhood are fully eaten.

- Scavenger fitness consists of the sum of the fraction of remains of the prey it eats in its local neighbor-hood. Each consumed prey adds *e^- ^*^(1-*fractioneaten*) ^to the fitness of the scavenger. Note that the approximation of scavengers is based on the original (x, y) of prey and that when there are no remains of a prey after a predator, scavengers can get no fitness on this prey.

### • selection

- apply to the prey, predator and scavenger present at position (i, j):

* add all fitness in local neighborhood to determine competition

* select replicator from neighborhood with a chance proportional to their fitness.

### • reproduction

1. Apply mutational operators on selected individuals with chance:

- *μ_prey _*per value for prey

- *μ *per element and *_GCR _*for predators and scavengers

2. let new individual inhabit cell

Simulations are stopped when either an individual solution (a predator coding for exactly the target function) has evolved, spread through the population and stayed in the population for 250 time steps, or if the maximum of 1500 generations is reached. An ecosystem based solution (the combination of a predator and scavenger exactly coding for the target function) is classified as either stable, when the solution stays in the population for the rest of the simulation or unstable if the solution is lost before the end of the simulation. A last possibility is an ecosystem based solution preceding an individual based solution as a transient state.

## Authors' contributions

Both authors conceived of the study and were involved in writing the manuscript. FK carried out most of the model work. PH supervised all aspects of the research.

All authors read and approved the final manuscript.
